# Global prevalence of barriers and facilitators to physical activity in children and adolescents: A systematic review with meta-analysis^☆^

**DOI:** 10.1016/j.pmedr.2025.103230

**Published:** 2025-09-10

**Authors:** Bruno Oliveira Amorim Sampaio, Armando Rodrigues de Alencar Santos, Marcus Vinícius Nascimento-Ferreira, Augusto César Ferreira De Moraes

**Affiliations:** a Department of Public Health, Faculty of Public Health, University of São Paulo, São Paulo 01246-904, SP, Brazil; b Postgraduate Program in Science and Health Education, Federal University of Tocantins, Tocantins 77001-090, TO, Brazil; c Federal University of Tocantins, Palmas 77650-000, Brazil; d The University of Texas Health Sciences Center at Houston, School of Public Health in Austin, Department of Epidemiology, Michael & Susan Dell Center for Healthy Living, Austin 78701, TX, USA

**Keywords:** Physical inactivity, Sedentary behavior, Lifestyle, Exercise, Youth

## Abstract

**Objectives:**

This study systematically reviewed the literature on the prevalence of barriers and facilitators to physical activity in children and adolescents and provided global prevalence estimates through meta-analysis.

**Methods:**

Articles published in PubMed (1966–), Scopus (1970–), and Web of Science (1900–) were searched from database inception through May 30, 2024. The search was conducted between May 15 and May 30, 2024. Sixteen studies were included, with 14 pooled for adolescents and 2 for children. The protocol was registered in PROSPERO (ID: CRD42024512962).

**Results:**

In children, the prevalence of barriers to physical activity ranged from 12 % to 26 %, with a pooled prevalence of 23 % (95 % CI, 19 %, 27 %). Facilitators in children ranged from 12 % to 33 %, with a pooled prevalence of 28 % (95 % CI, 24 %, 33 %). Among adolescents, barriers ranged from 3 % to 65 %, with a pooled prevalence of 32 % (95 % CI, 20 %, 44 %). Facilitators in adolescents ranged from 9 % to 21 %, with a pooled prevalence of 10 % (95 % CI, 8 %, 11 %).

**Conclusions:**

Barriers are more prevalent in adolescents, while facilitators are more prevalent in children. These findings highlight the need for targeted interventions and reveal research gaps, particularly regarding barriers in children and facilitators in adolescents.

## Highlights

• Adolescents face more barriers to physical activity than children.
• Children have more facilitators to engage in physical activity.
• Large variability in prevalence values due to study methodology.
• Research gap: barriers in children and facilitators in adolescents.
• Standardized tools are needed to improve data consistency.

## 1 Introduction

Physical activity is a complex phenomenon that encompasses various domains and varies according to age, sex, seasonality, day of the week, and the time of day it is performed. The importance of physical activity in children and adolescents is widely recognized for its benefits to physical, mental, and social health in this population (Bull et al., 2020; Guthold et al., 2020). For this reason, the World Health Organization (WHO) recommends that children and adolescents engage in at least 60 min of moderate to vigorous physical activity daily, preferably through aerobic activities (Bull et al., 2020). However, studies show that the pediatric population faces challenges in meeting this recommendation; approximately 80 % of adolescents worldwide are considered insufficiently active (Guthold et al., 2020; Marques et al., 2020; Sallis et al., 2016; Neil-Sztramko et al., 2021; Hallal et al., 2006; van Sluijs et al., 2021).

Adherence to the WHO recommendations for physical activity in children and adolescents requires consideration of both barriers and facilitators. Barriers are factors that hinder or prevent regular engagement in physical activity, including individual factors (e.g., lack of energy, motivation, or self-esteem), social factors (e.g., insufficient family support or encouragement), and environmental factors (e.g., adverse weather conditions or inadequate infrastructure) (Deforche et al., 2006; Sallis et al., 2000). These are commonly assessed using standardized questionnaires or scales, often employing Likert-type items (e.g., 1 to 5) to gauge the perceived intensity of each barrier (Allison et al., 1999; Brown, 2005). Facilitators, conversely, are factors that promote or enable participation in physical activity, representing the opposite construct of barriers (Beets et al., 2010; Edwardson and Gorely, 2010). They include individual factors (e.g., intrinsic motivation, awareness of health benefits), social factors (e.g., parental support, positive peer influence), and environmental factors (e.g., favorable conditions, access to appropriate spaces and equipment). Their measurement follows similar approaches to barriers, typically using Likert-type questionnaires to quantify perceived facilitators (Beets et al., 2010; Edwardson and Gorely, 2010; Davison and Jago, 2009).

No systematic review has previously assessed the prevalence of barriers and facilitators to physical activity among children and adolescents. This study aimed to systematically review the literature on the prevalence of barriers and facilitators in children and adolescents (aged 5–19 years, as defined by World Health Organization criteria) and to provide global prevalence estimates through a meta-analysis.

## 2 Methods

### 2.1 Identification of eligible studies – Electronic search and other sources

This systematic review followed the recommendations of the Preferred Reporting Items for Systematic Reviews and Meta-Analyses (PRISMA) methodology which include a checklist of 27 essential items to ensure transparent reporting (Moher, D., Liberati, A., Tetzlaff, J., Altman, D.G.; PRISMA Group, 2009). The study protocol was registered in the Prospective Register of Systematic Reviews (PROSPERO) under ID CRD42024512962. Searches were conducted in three electronic databases: PubMed (1966–), Scopus (1970–), and Web of Science (1900–) from database inception through May 30, 2024. The searches were performed between May 15 and May 30, 2024. No restrictions were placed on the publication year range. The search strategies were adapted for each database using specific descriptors and Boolean operators (“AND”,”OR”).

The search strategy included four groups of terms. The first group comprised child-related terms, including child, children, adolescence, and youth. The second group included perception-related terms such as perceived, barrier, barriers, facilitator, and facilitators. The third group contained activity-related terms, including physical activity, exercise, and physical exercises. The fourth group focused on prevalence-related terms, such as prevalence, prevalence studies, cross-sectional studies, and survey.

All descriptors were in English and derived from the Medical Subject Headings (MeSH) terms. An example of the search syntax used in PubMed was: [(child OR children OR adolescence) AND (barrier OR facilitator) AND (physical activity OR exercise) AND (prevalence OR cross-sectional studies)]. Filters applied included full-text availability, language (English, Portuguese, or Spanish), and article type. A flow diagram illustrates the selection process and the number of records retrieved.

### 2.2 Inclusion and exclusion criteria

Inclusion criteria comprised peer-reviewed original research articles, cross-sectional studies or baseline cohort studies, studies that assessed and reported the prevalence of perceived barriers and facilitators to physical activity, and studies that included at least one of the age groups of interest for the systematic review (5 to 19 years old). Studies were excluded if their samples were not composed of both sexes, if they did not report at least one barrier or facilitator, or if they were conducted with specific populations (e.g., obese, diabetic, etc.).

### 2.3 Assessment, data extraction, and analysis

The retrieved studies were imported into EndNote for reference management, and duplicates were removed. Studies were sequentially excluded based on titles, abstracts, and full-text reviews. Study selection was conducted independently and in a blinded manner by two researchers, with a third researcher involved to resolve any potential disagreements. For qualitative synthesis, data were extracted on publication information (first author, publication year, journal), study details (country, study year, sample size, participant age, proportion of female participants), methodology (type and structure of the questionnaire, including domains, number of questions, and response options), and identified barriers and facilitators. The extracted data were systematically recorded to facilitate comparison and synthesis across studies.

### 2.4 Statistical analysis

The prevalence of outcomes and their respective 95 % confidence intervals (95 % CI) were presented. The confidence intervals were extracted directly from the articles. For the studies that did not report prevalence estimates with corresponding 95 % confidence intervals, these parameters were manually calculated. When available, raw data on the number of individuals identified with barriers and/or facilitators (cases) and the total sample size were used to estimate prevalence and their respective 95 % confidence intervals. For this purpose, the command “cii proportion N n” was used, where *N* represents the total sample size and *n* represents the number of individuals reporting barriers and/or facilitators to physical activity practice.

In the meta-analysis, to determine which model to use, we performed the I^2^ heterogeneity test. This test indicates the percentage of total variability attributable to heterogeneity rather than to chance. The following classification was adopted: values between 0 and 25 % indicate low variability; between 26 and 50 % moderate variability; between 51 and 75 % high variability; and above 75 % suggest high variability among the study results (Higgins and Thompson, 2002). Since the I^2^ test values were high, we used the random-effects model, as there was an expectation of significant variation among the included studies, typically due to differences in sampling, measurement instruments used, methodologies, or the studied populations (Borenstein et al., 2011). The random-effects model is preferred over the fixed-effects model because, in reviews with high heterogeneity among studies, it provides wider confidence intervals, incorporating the variability of the observed effects (Schwarzer et al., 2015).

To represent the prevalences of barriers and facilitators from individual studies and globally, we used a Forest Plot. The global prevalence refers to the pooled prevalence derived from the studies included in the article. Subsequently, to detect publication bias, a Funnel Plot was used. The presence of asymmetry in the Funnel Plot suggests potential bias, as studies with smaller sample sizes and non-significant results tend to remain unpublished (Egger et al., 1997). The barriers and facilitators were identified in the studies through a thorough review of all questions included in each questionnaire analyzed. The questions were examined and grouped into domains of barriers and facilitators based on the responses used to define these domains. The statistical software used to perform all analyses presented in the article was STATA version 16.

## 3 Results

### 3.1 Study selection and data collection

**Fig. 1:**
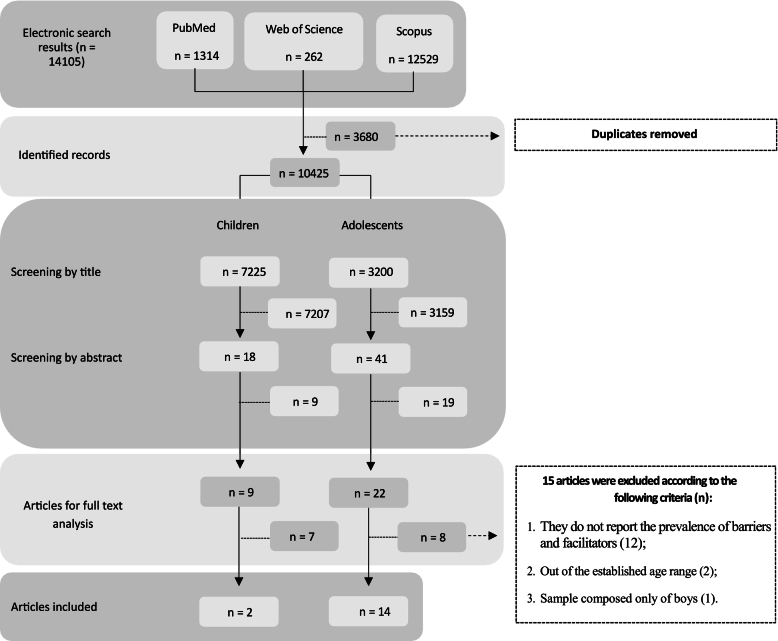
Selection process and number of articles included in the review.

Fig. 1 shows the selection process and the number of articles included in the review according to the PRISMA protocol (Moher, D., Liberati, A., Tetzlaff, J., Altman, D.G.; PRISMA Group, 2009). Initially, a total of 14,105 studies were identified in the databases. Of these, 3680 were duplicates, resulting in 10,425 studies screened. After reading the titles and abstracts, 31 studies were considered eligible and read in full. Fifteen studies were excluded after full-text reading. The reasons for exclusion were: studies that did not report the prevalence of barriers and facilitators for physical activity (*n* = 12); studies that used an age range that did not match the inclusion criteria (*n* = 2); and studies with samples composed only of boys (n = 1). Finally, 16 articles (0.1 % of the total) were included in the qualitative synthesis (Fig. 1). Of the 16 articles included in the qualitative analysis (Thompson et al., 2001; Santos et al., 2010; Youssef et al., 2013; Dias et al., 2015; Ramírez-Vélez et al., 2016; Jodkowska et al., 2017; Serrano et al., 2017; Payan et al., 2017; Ng et al., 2020; Camargo et al., 2021; Lazarowicz et al., 2021; Mawer et al., 2022; Silva et al., 2022; Koski et al., 2022; Mata et al., 2022; Nally et al., 2022), 14 were with samples composed of adolescents (Santos et al., 2010; Youssef et al., 2013; Dias et al., 2015; Ramírez-Vélez et al., 2016; Jodkowska et al., 2017; Serrano et al., 2017; Payan et al., 2017; Ng et al., 2020; Camargo et al., 2021; Lazarowicz et al., 2021; Mawer et al., 2022; Silva et al., 2022; Koski et al., 2022; Mata et al., 2022) and two articles with samples of children (Thompson et al., 2001; Nally et al., 2022). The year of publication of the studies varied between (2001) (Thompson et al., 2001) and (2022) (Mawer et al., 2022; Silva et al., 2022; Koski et al., 2022; Mata et al., 2022; Nally et al., 2022), with the year 2022 being the most prevalent, accounting for 31 % of the studies included for full-text reading.

Regarding the geographic distribution of the studies worldwide, seven studies were conducted in the Americas, with Brazil being the most represented, accounting for four studies (Santos et al., 2010; Dias et al., 2015; Camargo et al., 2021; Silva et al., 2022) and the United States with two (Thompson et al., 2001; Payan et al., 2017). In the European continent, six studies were conducted in six different countries, namely, Spain (Serrano et al., 2017), Finland (Koski et al., 2022), Ireland (Ng et al., 2020), Nothern Ireland (Nally et al., 2022), Poland (Jodkowska et al., 2017) and Portugal (Mata et al., 2022). In Oceania, two studies were conducted in Australia (Lazarowicz et al., 2021; Mawer et al., 2022). In Asia, one study was conducted in Oman (Youssef et al., 2013). No studies on this topic were selected from Africa. Table 1 provides a summary of the selected articles, including information on the first author, study country, year of publication, total number of participants, age range, and proportion of girls.

**Table 1:** Descriptive analysis of the studies analyzed.

First Author	Country	Year of publication	*n* total study	Average age	Proportion of girls (%)
Thompson et al	United States	2001	338	Not Reported	51.1
Santos et al	Brazil	2010	1609	Not Reported	59.6
Youssef et al	Oman	2013	439	Not Reported	51.2
Dias et al	Brazil	2015	1409	16.1 ± 0.5	54.9
Ramírez-Velez et al	Colombia	2016	5663	20.3 ± 2.1	40.8
Jodkowska et al	Poland	2017	2015	14.9 ± 1.2	54.7
Serrano et al	Spain	2017	248	15.3 ± 1.8	51.6
Payán et al	United States	2019	64	16.3 ± 1.3	67.2
Ng et al	Ireland	2020	1214	Not Reported	72.0
Camargo et al	Brazil	2021	1518	Not Reported	59.2
Lazarowicz et al	Australia	2021	5001	13 ± 1.1	25.8
Mawer et al	Australia	2022	48	19.5 ± 2.7	63.0
Silva et al	Brazil	2022	119	15.9 ± 0.9	53.0
Koski et al	Finland	2022	2728	Not Reported	50.9
Mata et al	Portugal	2022	1369	14.4 ± 1.7	54.6
Nally et al	Nothern Ireland	2022	50	Not Reported	56.0

Table 2 presents information regarding the questionnaires used by the studies to measure barriers and facilitators. Different questionnaires were used for this purpose, such as the BBAQ (Barriers to Being Active Quiz) (Ramírez-Vélez et al., 2016; Jodkowska et al., 2017), BPAQ (Barriers to Physical Activity Questionnaire) (Silva et al., 2022) and the EPC (Escala de Percepción de Barreras) (Serrano et al., 2017), in addition to other questionnaires that were unnamed and were either created or adapted by the authors of the article or by expert committees for the purpose of the study.

**Table 2:** Summary of studies and questionnaires assessing barriers and facilitators to physical activity in children and adolescents.

First Author	Sample	Questionnaire name	Number of questions	Response options	Domain of Barriers	Domain of Facilitators
Thompson	Children	Knowledge, Attitudes, and Behaviors Survey	10	Not Reported	Lack of willpower/Lack of energy/Social influence/Weather/Lack of resources	Absent
Nally	Children	Focus Group	Not Reported	Not Reported	Weather/Lack of time/Lack of skill/Lack of resources/Fear of injury/Lack of spaces	Social support/Environmental factors
Mawer	Adolescents	Not Reported	Not Reported	Not Reported	Weather/Social influence/Lack of skill/Lack of time/Lack of resources	Social support/Health improvement/Enjoyment of physical activity/Appropriate place for physical activity
Jodkowska	Adolescents	Barriers to Being Active Quiz	21	Likert Scale	Lack of energy/Lack of time/Social influence/Lack of willpower/Lack of skill/Lack of resources	Absent
Silva	Adolescents	Barriers to Being Active Quiz	12	Dichotomous	Lack of energy/Lack of time/Social influence/Lack of willpower/Lack of skill/Lack of resources/Weather	Absent
Ramírez-Vélez	Adolescents	Barriers to Being Active Quiz	21	Likert Scale	Lack of energy/Lack of time/Social influence/Lack of willpower/Lack of skill/Lack of resources	Absent
Payán	Adolescents	Not Reported	Not Reported	Dichotomous	Lack of motivation/Lack of time/ Lack of safety	Absent
Youssef	Adolescents	Not Reported	12	Likert Scale	Lack of skill/Lack of energy/Social influence/Lack of resources	Absent
Santos	Adolescents	Not Reported	12	Likert Scale	Lack of energy/Lack of time/Social influence/Lack of willpower/Lack of skill/Lack of resources/Weather	Absent
Koski	Adolescents	Not Reported	18	Likert Scale	Individual barriers/Social barriers/Environmental barriers	Absent
Mata	Adolescents	Not Reported	22	Not Reported	Lack of energy/Lack of time/Social influence/Lack of willpower/Lack of skill/Lack of resources/Weather	Absent
Camargo	Adolescents	Not Reported	12	Likert Scale	Lack of energy/Lack of time/Social influence/Lack of willpower/Lack of skill/Lack of resources/Weather	Absent
Lazarowicz	Adolescents	Not Reported	13	Not Reported	Lack of energy/Lack of time/Social influence/Lack of willpower/Lack of skill/Lack of resources/Weather	Absent
Ng	Adolescents	Not Reported	Not Reported	Likert Scale	Coronavirus/Lack of motivation/Lack of resources/Lack of routine/Health problems/Mental health/Closed institutions	Coronavirus/More time to exercise/No classes/Health improvement/Nothing to do/Needed to leave the house/Taking walks
Serrano	Adolescents	Escala de Percepción de Barreras	17	Likert Scale	Lack of skill/Lack of time/Lack of willpower/Lack of safety	Absent
Dias	Adolescents	Not Reported	12	Likert Scale	Lack of energy/Lack of time/Social influence/Lack of willpower/Lack of skill/Lack of resources/Weather	Absent

The number of questions in the questionnaires ranged from 10 to 22, and the most prevalent response option was the Likert scale, which was present in 56 % of the studies (Santos et al., 2010; Youssef et al., 2013; Dias et al., 2015; Ramírez-Vélez et al., 2016; Jodkowska et al., 2017; Serrano et al., 2017; Ng et al., 2020; Camargo et al., 2021; Koski et al., 2022), followed by dichotomous response options, present in 12 % of the studies (Payan et al., 2017; Silva et al., 2022). The remaining studies (32 %) did not report which response option was used (Thompson et al., 2001; Lazarowicz et al., 2021; Mawer et al., 2022; Mata et al., 2022; Nally et al., 2022). Regarding the domains of barriers to physical activity, they were very similar across the studies, with key barriers including lack of willpower, lack of energy, social influence, weather, lack of resources, lack of time, and lack of skill. Only one study classified the domains into social barriers, environmental barriers, and individual barriers (Koski et al., 2022). Regarding the domains of facilitators for physical activity, only four studies reported them (Thompson et al., 2001; Ng et al., 2020; Mawer et al., 2022; Nally et al., 2022), examples of facilitators reported include improvements in health, enjoyment of physical activity, access to appropriate locations for physical activity, more time to engage in physical activity, no classes, having nothing else to do, and the need to leave the house to go for a walk.

Table 3 presents the prevalence of barriers and facilitators to physical activity in children and adolescents. For children, the prevalence of barriers ranged from (12 %) (Nally et al., 2022) and (26 %) (Thompson et al., 2001) and the global prevalence of barriers was 23 % (95 % CI = 19 %, 27 %). The prevalence of facilitators for physical activity in children ranged from (12 %) (Nally et al., 2022) and (33 %) (Thompson et al., 2001) and the global prevalence of facilitators was 28 % (95 % CI = 24 %, 33 %).

**Table 3:** Prevalence of Barriers and Facilitators for Physical Activity Practice in Children and Adolescents.

**First author**	**Prevalence of Barriers (CI 95 %)**	**Prevalence of Facilitators (CI 95 %)**	**Global prevalence of barriers (CI 95 %)**	**Global prevalence of facilitators (CI 95 %)**	**I**^**2**^**- Heterogeneity (%)**
Thompson	26 (22,31)	33 (29, 39)	Children = 23 (19, 27)	Children = 28 (24, 33)	Barriers in Children = 86 Facilitators in Children = 93
Nally	12 (6, 24)	12 (6, 24)			
Mawer	27 (17, 41)	21 (12, 34)	Adolescents = 32 (20, 44)	Adolescents = 10 (8, 11)	Barriers in Adolescents = 99.7 Facilitators in adolescents = 72.8
Jodkowska	40 (38, 42)	Not Reported			
Silva	54 (45, 62)	Not Reported			
Ramírez-Vélez	65 (64, 66)	Not Reported			
Payán	39 (28, 51)	Not Reported			
Youssef	53 (48, 57)	Not Reported			
Santos	28 (26, 30)	Not Reported			
Koski	10 (9, 11)	Not Reported			
Mata	8 (6, 9)	Not Reported			
Camargo	32 (30, 34)	Not Reported			
Lazarowicz	19 (18, 20)	Not Reported			
Ng	3 (2, 4)	9 (8, 11)			
Serrano	31 (25, 37)	Not Reported			
Dias	39 (37, 42)	Not Reported			

The prevalence of barriers to physical activity in adolescents ranged from (3 %) (Ng et al., 2020) and (65 %) (Ramírez-Vélez et al., 2016) and the global prevalence of barriers was 32 % (95 % CI = 20 %, 44 %). The prevalence of facilitators for physical activity in adolescents ranged from (9 %) (Nally et al., 2022) and (21 %) (Mawer et al., 2022) and the global prevalence of facilitators was 10 % (95 % CI = 8 %, 11 %).

It was found from the I^2^ test that there is very high heterogeneity among the studies for both children and adolescents (greater than 75 %), even after opting for the random-effects model for the meta-analysis. Therefore, we created the Funnel Plot to help identify whether part of this heterogeneity is due to publication bias or study size, as publication bias tends to distort the funnel (producing an asymmetric shape) and may affect the pooled estimate and, consequently, the heterogeneity test (Higgins and Thompson, 2002; Egger et al., 1997; Rücker et al., 2011). Additionally, if the Funnel Plot shows a concentration of small studies with extreme estimates, this may indicate that the high I^2^ is partially driven by variability in the estimates from smaller studies, which often exhibit greater statistical variability (Higgins and Thompson, 2002; Egger et al., 1997; Rücker et al., 2011).

The forest plot for the prevalence of barriers to physical activity in adolescents (Supplementary Material A) indicated that studies with larger sample sizes had greater influence on the pooled prevalence estimate than those with smaller samples. Seven studies had prevalence estimates falling within the 95 % confidence interval of the pooled estimate (Santos et al., 2010; Dias et al., 2015; Jodkowska et al., 2017; Serrano et al., 2017; Payan et al., 2017; Camargo et al., 2021; Mawer et al., 2022). Among them, four studies had sample sizes exceeding 1000 adolescents (Santos et al., 2010; Dias et al., 2015; Jodkowska et al., 2017; Camargo et al., 2021), while three had fewer than 500 participants (Serrano et al., 2017; Payan et al., 2017; Mawer et al., 2022). The other seven studies presented prevalence estimates outside the CI of the global prevalence (Youssef et al., 2013; Ramírez-Vélez et al., 2016; Ng et al., 2020; Lazarowicz et al., 2021; Silva et al., 2022; Koski et al., 2022; Mata et al., 2022); five of these were large-sample studies (Ramírez-Vélez et al., 2016; Ng et al., 2020; Lazarowicz et al., 2021; Koski et al., 2022; Mata et al., 2022) and two had small samples (Youssef et al., 2013; Silva et al., 2022). Although the funnel plot showed no evidence of asymmetry, studies with small samples accounted for a significant proportion (34 %) of the overall weight in the pooled estimate (Youssef et al., 2013; Serrano et al., 2017; Payan et al., 2017; Mawer et al., 2022; Silva et al., 2022).

The forest plot for the prevalence of facilitators of physical activity in adolescents (Supplementary Material B) revealed that one study with a large sample size (Ng et al., 2020) accounted for 98 % of the weight in the pooled estimate, while the smaller study (Mawer et al., 2022) contributed only 2 %. The prevalence estimate from study (Mawer et al., 2022) fell on the lower boundary of the pooled CI and displayed a wide confidence interval due to its limited sample size. Despite this, the funnel plot showed no evidence of asymmetry.

For children, the forest plot for the prevalence of barriers to physical activity (Supplementary Material C) demonstrated that the study with the larger sample size (Thompson et al., 2001) contributed 78 % to the pooled prevalence, while the smaller study (Nally et al., 2022) accounted for 22 %. Both studies had prevalence estimates within the pooled 95 % CI, although one of them (Nally et al., 2022) was positioned at the margin of the confidence interval. No asymmetry was identified in the funnel plot.

Similarly, in the analysis of facilitators to physical activity in children (Supplementary Material D), the study with the larger sample size (Thompson et al., 2001) contributed 76 % to the pooled estimate, compared to 24 % from the smaller study (Nally et al., 2022). While the prevalence from study (Thompson et al., 2001) was contained within the CI of the pooled estimate, the estimate from study (Nally et al., 2022) was outside that interval. Although no asymmetry was observed in the funnel plot, a notable precision error was evident in the estimate from the smaller study (Nally et al., 2022).

## 4 Discussion

### 4.1 Barriers and facilitators to physical activity in children

Our systematic review identified only two studies addressing barriers and facilitators to physical activity in children, revealing a substantial gap in the literature. Notably, a 21-year gap separates these studies, highlighting the limited attention given to this age group. The global prevalence estimates for barriers and facilitators were heavily influenced by the study conducted by Thompson et al (Thompson et al., 2001). which had a sample size 6.7 times larger than that of Nally et al (Nally et al., 2022). This imbalance underscores the importance of larger, representative studies in obtaining reliable global estimates (Thompson et al., 2001).

The measurement instruments used in the two studies differed significantly. Thompson et al. employed a structured questionnaire, while Nally et al (Thompson et al., 2001). utilized focus groups (Nally et al., 2022). This methodological disparity introduces potential biases related to instrument reliability, validity, and generalizability. For example, reliability ensures consistent measurement across contexts and samples, yet differing methods may reflect more on instrument characteristics than the phenomenon being studied (Field, 2018). Similarly, validity issues arise when instruments with distinct scales or approaches fail to adequately capture the intended construct (Kline, 2016). Without standardized tools validated across diverse populations, the reproducibility and applicability of findings remain compromised (Jodkowska et al., 2015). Future research should prioritize the development and use of psychometrically robust instruments to enhance comparability and generalizability across studies.

The wide confidence intervals reported by both studies for both barriers and facilitators also caught our attention. A large confidence interval indicates high variability in the data and low precision in the estimate of the measure, which can undermine the interpretation and utility of the results. Wide confidence intervals typically reflect a small sample size, inconsistencies in responses, or a questionnaire with reliability issues. These factors compromise the ability to generalize the results to the target population and reduce the accuracy of the inferences (Cohen, 2013; Field, 2018; Kline, 2016).

### 4.2 Barriers and facilitators to physical activity in adolescents

The search in our systematic review selected 14 articles that reported the prevalence of barriers and facilitators to physical activity in children and adolescents (Santos et al., 2010; Youssef et al., 2013; Dias et al., 2015; Ramírez-Vélez et al., 2016; Jodkowska et al., 2017; Serrano et al., 2017; Payan et al., 2017; Ng et al., 2020; Camargo et al., 2021; Lazarowicz et al., 2021; Mawer et al., 2022; Silva et al., 2022; Koski et al., 2022; Mata et al., 2022). This number was seven times higher than that found for children in our systematic review. All 14 articles reported the prevalence of barriers, but only two of them also reported the prevalence of facilitators (Ng et al., 2020; Mawer et al., 2022). We observed that studies on this topic began to be published in (2010) (Santos et al., 2010) and continued to be published steadily until (2022) (Silva et al., 2022), demonstrating that the literature has given more attention to barriers and facilitators for physical activity in adolescents compared to children.

There were seven studies in which the reported prevalence of barriers was contained within the confidence interval of the global barriers estimate (Santos et al., 2010; Dias et al., 2015; Jodkowska et al., 2017; Serrano et al., 2017; Payan et al., 2017; Camargo et al., 2021; Mawer et al., 2022). Of these seven studies, it is important to highlight that the three studies that most closely approximated the global prevalence estimate were conducted in Brazil (Santos et al., 2010; Dias et al., 2015; Camargo et al., 2021). These studies had large sample sizes, ranging between (1409) (Dias et al., 2015) and (1609) (Santos et al., 2010) adolescents, used the same questionnaire developed in the study by Santos et al (Santos et al., 2010), which was validated for the Brazilian adolescent population, consisting of 12 questions and using the Likert scale as the response option. Next, we have the study conducted in Poland (Jodkowska et al., 2017), with a sample of 2015 adolescents, who used the BBAQ questionnaire, which is validated for Polish (Jodkowska et al., 2015), Colombian (Rubio-Henao et al., 2015) and Brazilian (Sampaio et al., 2024) adolescents. The BBAQ consists of 21 questions and uses the Likert scale as the response option. Finally, we have the study conducted in Spain (Serrano et al., 2017), with a sample of 248 adolescents, this study used the EPC questionnaire, validated for Spanish adolescents, consisting of 17 questions and utilizing the Likert scale as the response option. The other two studies employed the focus group strategy (Payan et al., 2017; Mawer et al., 2022). It is important to highlight that seven studies were not within the confidence interval of the global barriers estimate (Youssef et al., 2013; Ramírez-Vélez et al., 2016; Ng et al., 2020; Lazarowicz et al., 2021; Silva et al., 2022; Koski et al., 2022; Mata et al., 2022).

Regarding facilitators for physical activity in adolescents, two studies reported the prevalence of facilitators (Ng et al., 2020; Mawer et al., 2022). Only the study conducted in Ireland was within the confidence interval of the global prevalence estimate for facilitators in adolescents (Ng et al., 2020).This study had a sample of 1214 adolescents, and the questionnaire used was created by the authors of the article, with the Likert scale as the response option. Due to its much larger sample size compared to the study conducted in Australia, it contributed more significantly to the global prevalence estimate for facilitators (Mawer et al., 2022), which had a sample of 48 adolescents and used the focus group strategy, the study (Ng et al., 2020) contributed 98 % to the global prevalence estimate of facilitators for adolescents. Finally, we highlight the low number of studies conducted worldwide on this topic.

### 4.3 Strengths and limitations of the study

The main strength of the article lies in its novelty, being the first study in the literature to estimate the global prevalence of barriers and facilitators to physical activity in children and adolescents. A careful selection of articles was conducted, following an already established search protocol for systematic reviews. Furthermore, a specific statistical analysis was performed for a phenomenon of interest with heterogeneous values.

Regarding the study's limitations, we found only two articles reporting the prevalence of barriers and facilitators for physical activity in children and two articles reporting the prevalence of facilitators for adolescents. This leads to limitations such as difficulties in assessing the heterogeneity of the results, disproportionate impact from the weight of each study, and limited interpretation of the findings.

## 5 Conclusion

This review highlights critical insights into the global prevalence of barriers and facilitators for physical activity participation among children and adolescents. The findings indicate that adolescents face a higher prevalence of barriers to physical activity compared to children, whereas children benefit from a higher prevalence of facilitators for physical activity relative to adolescents. The variability in reported prevalence values across studies is marked, driven by methodological inconsistencies, including the absence of standardized measurement tools, differences in response options among instruments, variability in sample sizes, and the lack of precision in some questionnaires. Moreover, a notable gap in the literature exists regarding studies that focus specifically on barriers to physical activity in children and facilitators in adolescents. These results emphasize the need for standardized methodologies and targeted research to enhance the understanding of the factors influencing physical activity behaviors in these populations, which is crucial for the development of effective intervention strategies.

## CRediT authorship contribution statement

**Bruno Oliveira Amorim Sampaio:** Writing – review & editing, Writing – original draft, Methodology, Investigation, Formal analysis, Data curation, Conceptualization. **Armando Rodrigues de Alencar Santos:** Writing – review & editing, Investigation, Formal analysis, Data curation. **Marcus Vinícius Nascimento-Ferreira:** Writing – review & editing, Validation, Supervision. **Augusto César Ferreira De Moraes:** Writing – review & editing, Writing – original draft, Methodology, Investigation, Formal analysis, Data curation, Conceptualization.

## Funding source

This study was financed in part by the Coordenação de Aperfeiçoamento de Pessoal de Nível Superior - Brasil (CAPES) - Finance Code 001. Augusto César Ferreira De Moraes and Marcus Vinícius Nascimento-Ferreira received funds from the IC2 Institute of The University of Texas at Austin.

## Declaration of competing interest

The authors declare that they have no known competing financial interests or personal relationships that could have appeared to influence the work reported in this paper.

## Appendix A Supplementary data

Supplementary material Image 1
