# Supplementary material for: Global prevalence of barriers and facilitators to physical activity in children and adolescents: A systematic review with meta-analysis
Source: Prev Med Rep. 2025 Sep 10;58:103230. doi: 10.1016/j.pmedr.2025.103230 (PMC12491723; doi:10.1016/j.pmedr.2025.103230)
Supplement: Supplementary file 1 — Supplementary material [file mmc1.docx]

**Supplementary Material A: Forest Plot of the prevalence of barriers to physical activity in adolescents**


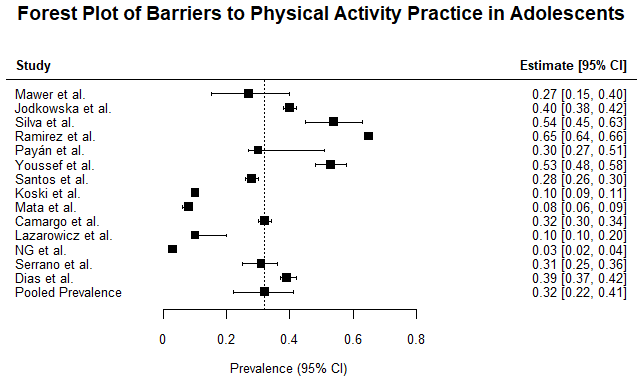


**Supplementary Material B: Forest Plot of the prevalence of facilitators of physical activity in adolescents**


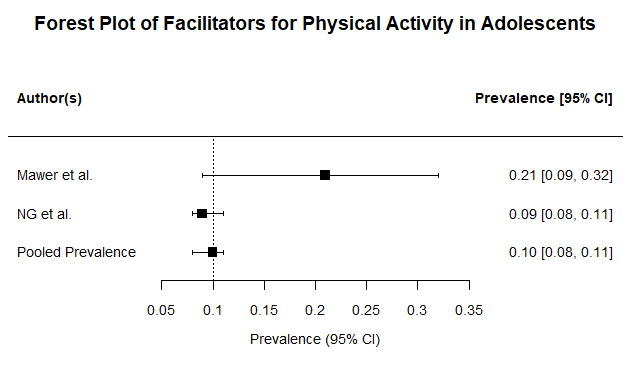


**Supplementary Material C: Forest Plot of the prevalence of barriers to physical activity in children**


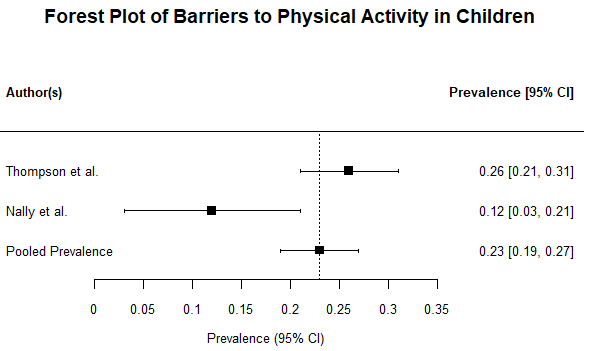


**Supplementary Material D: Forest Plot of the prevalence of facilitators of physical activity in children**


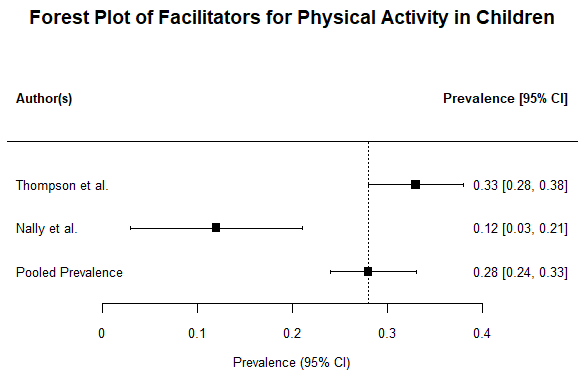


# Supplementary Material E: PRISMA 2020 Checklist – Systematic Review with Meta-analysis

| Item No. | PRISMA 2020 Checklist Item | Status | Comments |
| --- | --- | --- | --- |
| 1 | Title identifies the report as a systematic review. | Yes | The title includes "systematic review with meta-analysis". |
| 2 | Structured abstract following PRISMA for Abstracts. | Yes | Includes objectives, methods, results, and conclusions. |
| 3 | Rationale provided in context of what is known. | Yes | Clearly describes the literature gap. |
| 4 | Explicit objectives stated (e.g., based on PICO). | Yes | Population: 5–19 years; Outcome: prevalence; Exposure: barriers/facilitators. |
| 5 | Review protocol registered. | Yes | PROSPERO ID: CRD42024512962. |
| 6 | Eligibility criteria specified. | Yes | Detailed inclusion and exclusion criteria in Methods section. |
| 7 | Information sources and date of last search. | Yes | Databases and search date (May 30, 2024) reported. |
| 8 | Full search strategy for all databases. | Partial | Strategy described, but not fully reproducible or included as appendix. |
| 9 | Study selection process. | Yes | Two independent reviewers; process detailed clearly. |
| 10 | Data collection process. | Yes | Described clearly in Methods. |
| 11 | Data items specified. | Yes | Study, sample, instrument, prevalence, etc. reported. |
| 12 | Risk of bias in individual studies. | No | No formal assessment reported. |
| 13 | Outcomes and prioritization. | Yes | Prevalence of barriers and facilitators. |
| 14 | Synthesis methods (meta-analysis, heterogeneity, etc.). | Yes | STATA, random effects, I² described. |
| 15 | Reporting bias assessment (e.g., Funnel Plot). | Yes | Funnel plot used to assess publication bias. |
| 16 | Subgroup/sensitivity analyses. | Partial | Discussed sample size differences, but no formal subgroup analysis. |
| 17 | Study selection results with flow diagram. | Yes | PRISMA flow diagram included (Figure 1). |
| 18 | Study characteristics. | Yes | Provided in Table 1 and described in text. |
| 19 | Risk of bias across studies. | No | Not assessed using standard tools (e.g., JBI, NOS). |
| 20 | Results of individual studies. | Yes | Detailed in text and Table 3. |
| 21 | Results of syntheses (meta-analysis). | Yes | Pooled prevalence estimates with CI presented. |
| 22 | Presentation of results using plots (e.g., Forest Plot). | Yes | Forest and Funnel Plots presented. |
| 23 | Discussion of results with context. | Yes | Comparative discussion with prior studies. |
| 24 | Limitations of included studies. | Yes | Described limitations of studies and instruments. |
| 25 | Limitations of review process. | Yes | Acknowledged limitations of search and synthesis. |
| 26 | Funding and competing interests. | Yes | No funding/conflict information included. |
| 27 | Availability of data, code, or materials. | Yes | The data and statistical code used in this review are available |
